# Lifetime Analysis of Dies Manufactured by Conventional Processes and Reconditioned by Deposition Welding Operation

**DOI:** 10.3390/ma17071469

**Published:** 2024-03-22

**Authors:** Daniela Maria Iovanas, Adela-Eliza Dumitrascu

**Affiliations:** 1 Department of Materials Engineering and Welding, Transilvania University of Brasov, 1 Colina Universitatii, 500036 Brasov, Romania; daniela.iovanas@unitbv.ro; 2 Department of Manufacturing Engineering, Transilvania University of Brasov, 5 Mihai Viteazul, 500036 Brasov, Romania

**Keywords:** lifetime analysis, forging dies, deposition welding, reconditioned dies, coated electrodes, maximum likelihood estimation method

## Abstract

The refurbishment of dies by the deposition welding of wear areas is an efficient and economical process. The aim of this study was to conduct a comparative analysis of the lifetimes of different types of dies for the manufacturing of wagon wheels. The analyzed dies were manufactured by conventional processes (Type I) and reconditioned through a deposition welding procedure using a dedicated electrode (Type II). The Anderson–Darling test was conducted to analyze the goodness of fit of the lifetime data specific to the die types. The maximum likelihood estimation method (MLE) with a 95% confidence interval (CI) was applied in order to estimate the lifetime distribution parameters. It was found that the lifetimes of type II dies were longer than those of type I dies. The mean time to failure (MTTF) recorded for reconditioned dies was 426 min, while the mean time to failure of dies manufactured by conventional processes was approximatively 253 min. In addition, an accentuated hazard rate for type I dies compared to type II dies was observed. The results of this analysis emphasized the fact that dies can be restored to their initial operating capacity by successfully using deposition welding procedures that confer a high resistance to operational loads. At the same time, the use of these procedures allows for the sustainable development of resources and waste management.

## 1. Introduction

Transport is an essential element for the economic development of any country. Today, an increasing number of people, goods, and material goods are transported around the world. In this context, rail transport has an important place in today’s society due to a multitude of advantages. It provides the following: a high volume of transport, low energy and fuel consumption, safety, low pollutant emissions, etc. [1].

Rail transport is carried out using specialized vehicles on appropriate infrastructure, and an important role in ensuring transport is played by wagons that are specialized for the type of transport, cargo, or passengers. They move on a running track by means of wheels, which have the role of supporting the wagon, as well as rolling. Creating them involves high-cost steels and manufacturing processes. These processes include cold or hot plastic deformation, where the essential influencing factor in their productivity, and simultaneously the most requested element, is the die, which is made of alloy steels [2].

In the current context, in which the world’s population is growing, the demands for products are increasing, and the world economy is facing an urgent crisis of raw materials, materials, and energy—the need to significantly reduce consumption is becoming increasingly important. The investigation by [3] highlighted the potential benefits and opportunities associated with remanufacturing with respect to welding repair technologies, remaining useful life evaluation methods, and reliability analyses. The industrial repair strategies of the main types of dies were examined. Remanufacturing has attracted a lot of attention due to its ecological and economic advantages over the manufacture of new products [3]. Starting from these premises, attention has been directed toward to the manufacturing of more reliable products that involve dies that can be reused, modernized, and repaired, which leads to the development of modern repair technologies that are based on the reconditioning of products by applying deposition welding procedures in areas subject to operational wear.

Aspects of reconditioning through welding processes by deposition that are theorized in research are carried out in the field and reported in technical papers [4], which have described applying the research to cutting knives, as well as hammers for the mining industry and automotive parts. Reconditioning knives via welding deposition using modulated and interchangeable elements of a bimetal type, where the layers’ depositions are made of special filler materials of a tubular wire type, enhances their reliability; in addition, the costs of manufacturing, exploitation, and maintenance are significantly reduced [5]. Endurance evaluations of the deposited layers on coal hammer mills have proven the beneficial role of active surfaces with a hardness between 473 and 644 HV_5_ [6]. From the perspective of the metallographic analysis conducted by [7], comparisons between shielded metal arc welding (SMAW) and welding in gas (WIG) reconditioning processes have shown that the method of depositing the layers and the procedure used have a significant influence on the quality of the deposited layers (i.e., there is a low risk of defects occurring and better control of the heat input in the reconditioned parts). Also, the analysis of the hardness made by [8] demonstrated that the deposited material layer has a much greater hardness value than the base material, thereby increasing the resistance to wear.

Quality, along with reliability, represents the prerogative of cutting-edge technologies through competitiveness. However, the expansion of manufacturing technologies under globalization requires the use of new resources, which is in accordance with the strategies regarding the responsible use of resources, and that threatens the maintenance of quality and reliability.

Dies, due to operational demands, may be taken out of use as a result of major defects during the production process. Through applying finite element analysis (FEM) on hot forging dies, a wear coefficient was proposed by [9]. In addition, the process variables of temperature, pressure, velocity, and time were used to predict wear by [10]. Meanwhile, the effects of increased temperature and time on the properties of the surface layer of forging tools were studied by [11]. 

When analyzing the main types of damage on a pair of industrial dies for hot forging, the following types of wear were detected on the die surfaces: abrasion, mechanical, and thermal fatigue [12]. The phenomena related to wear mechanisms that appear in forging molds were also examined in [13]. In order to improve the mechanical characteristics of forged parts (deformability, strength, and toughness), studies have focused on the micro-alloy element content and on processing parameters [14], and excellent mechanical and structural properties have been obtained.

When taking into account the high cost of the materials from which dies are made, attention has been directed toward innovative reconditioning technologies, expensive manufacturing processes, and the economic implications of their premature removal from use and the resumption of operation [15].

The reliability approach represents an appropriate methodology for the lifetime and defect analysis of dies. This involves pairing an old concept and a new discipline. Testing for reliability encompasses a wide range of problems in all stages of a product’s life cycle, such as conception, design, manufacturing, execution, operation, etc. Today, residual life evaluations and reliability analyses, though well researched in other fields, are still immature within the remanufacturing of dies [3].

Reliability can be defined as a set of characteristics as follows [16]:

- From a qualitative point of view: the ability of the system to be used under specified times and conditions for as long a period as possible;
- From a qualitative point of view: the measure of the probability of a system functioning well according to specified requirements;
- Concerning the industrial system’s operational safety: if quality represents the totality of the product’s properties that make it suitable for a specific use, reliability indicates the product quality extended over time during usage.

The research in the field demonstrates that the most efficient and economical procedure for product wear area refurbishment is welding deposition [17] with wear protection systems [18]. Additional materials can also be used to increase the wear resistance of a die’s active surface through welding layers with high hardness [19,20,21], which protect the base metal. That procedure was applied in this study to manufacturing wagon wheel dies. To assess the benefits of its use, lifetime evaluation and reliability analysis were conducted [3]. 

Deposition welding procedures’ application leads to significant material savings by reconditioning dies instead of using other new dies made by conventional methods. In accordance with European strategies, these manufacturing processes are in keeping with goals of implementing sustainable waste management and sustainable development of used resources. 

From the multitude of welding processes that are currently applied in all fields (with electric arc with covered electrode, in protective gas environment, TIG, MIG-MAG, plasma, laser, etc.), and especially the melting ones, the electric arc welding process has been chosen, which is based on a special coated electrode that is especially designed and made. In this study, it was used for reconditioning defective dies designed for wagon wheel manufacturing, which was achieved through welding deposition. The aim of this paper is to apply inferential statistics to analyze the lifetimes expressed by the main reliability indices of two types of dies in our study.

## 2. Materials and Methods

### 2.1. Reconditioning by Deposition Welding of Dies Used for Wagon Wheels’ Manufacturing

The dies under study in this case are made of steels alloyed with Cr and Ni, hardened and tempered to a hardness of approximatively 35 HRC, which must provide high resistance at normal and high temperatures, high toughness, resistance to thermal fatigue, and resistance to hot wear and superficial oxidation [16].

**Figure 1:**
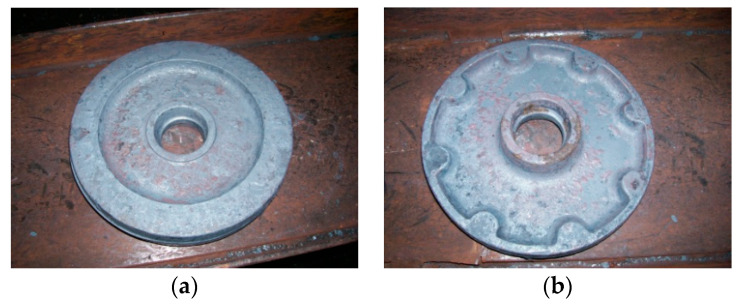
Half dies for obtaining wagon wheels: (**a**) upper half die; (**b**) lower half die.

Currently, for wagon wheel manufacturing, two half dies are used (Figure 1). During operation, the dies are subject to stresses with a high degree of triaxiality and severe abrasion, combined with corrosion, adhesion wear, and thermomechanical fatigue with radial and uniform distributions [16].

**Figure 2:**
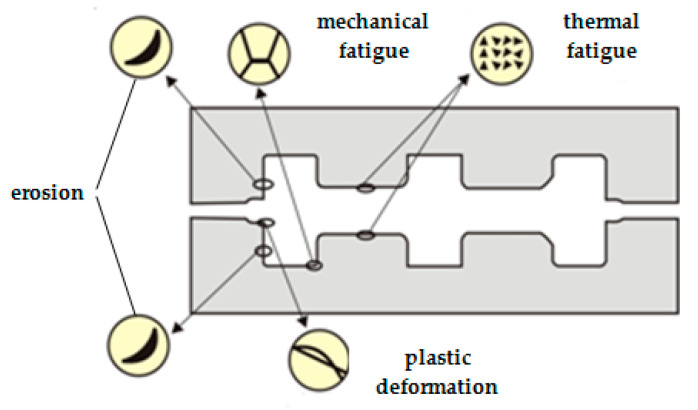
Phenomena that may occur during forging [16].

The most frequent phenomena that occur during the exploitation of half dies are shown in Figure 2 [16]:

- Erosion produced by the friction that occurs between the mold and the warm semi-finished product, covered by hard oxides;
- Thermal fatigue caused by successive expansions and contractions produced by the temporary contact between the mold and the hot semi-finished product;
- Mechanical fatigue produced by the repeated dynamic loads that occur during exploitation;
- Plastic deformations caused by the decrease in the hardness of the mold and in the yield point after heating.

**Figure 3:**
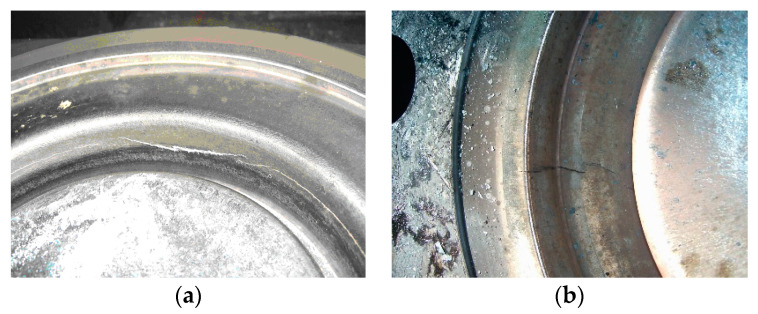
Defects arising in exploitation: (**a**) exfoliation, oxides, erosion; (**b**) cracks.

At the level of the active surfaces, these stresses cause defects such as circular and radial cracks, shape changes due to creep, and metal volume loss (Figure 3), with the possibility of removal from use.

This research was carried out on a sample of six half dies used for wagon wheel manufacturing, made of steel alloy type 55MoCrNi16 (STAS 3611:88). The analyzed wears were those generated predominantly in the working area: cracks, fissures, and detachments of oxidized and damaged material that led to their removal from use, after a relatively small number of wheels were produced.

Considering the large dimensions of these dies, for the reconditioning process, manual welding was chosen, using coated electrodes on the wagon wheel manufacturing line. The electrodes were specially designed and developed, as has been the subject of some research [19]. To increase wear resistance, they efficiently apply deposits with a hardness of approx. 45–60 HRC.

In order to develop the deposition technology and study the welded area’s properties, weld beads were deposited with the experimental electrode on plates with the same base material (BM) as the dies [16,22].

High weathering resistance was obtained by using alloyed electrodes, where the upper coatings were prepared with the necessary hardness to protect the base metal.

A coated electrode was designed for deposition by electric arc welding based on iron, with additions of chromium, tungsten, vanadium, and lanthanides. The material was developed in the form of a coated electrode, that is, an unalloyed metal rod with a coating containing alloying elements and lanthanides. The beads deposited by electric arc welding created an iron-based alloy with the following alloying elements: carbon 0.2–0.5%, silicon 1–1.5%, chromium 2–3%, vanadium 0.4–1%, and tungsten 3–5.5%, symbolized EiCr2.5W4.5 V [16]. The alloy had a maximum hardness of 45HRC (in the welded state). Deposition welding was carried out considering the parameters presented in Table 1.

The chemical composition of the deposition metal was determined using a Spetromax optical emission spectrometer and is presented in Table 2.

### 2.2. Statistical Analysis

Probabilistic analysis consists of a mix of measures and probability theories, discrete and continuous random variables, and their probable and expected properties [23,24,25].

There are several statistical methods that can be used to fit models and estimate failure rates (Kaplan–Meier approach, probability plotting, hazard plotting, graphical estimation, least squares, and maximum likelihood estimation) [26,27,28]. An appropriate statistical model—the Anderson–Darling goodness-of-fit test—was identified to determine whether a sample originated from a population with a specific distribution.

A statistical analysis of the estimated reliability indices was carried out using the Minitab 17 software (Minitab LLC, State College, PA, USA).

Given a continuous random variable *X*, the following is specified [29]:

- *f*(*x*) is the probability density function (PDF);
- *F*(*x*) is the cumulative density function (CDF).

If *X* is a continuous random variable, then the probability density function of *X* is a function *f*(*x*) such that for two numbers, *a* and *b*, with *a ≤ b* [29],

(1) $$P\left(a\leq X\leq b\right)=\int_{a}^{b}f\left(x\right)dx.$$

The cumulative distribution function is a function *F*(*x*) of a random variable *X* and is defined for a number *x* by [29]

(2) $$F\left(x\right)=P\left(X\leq x\right)=\int_{0,-\infty }^{x}f\left(x\right)dx.$$

The Anderson–Darling test consists of the following calculus of statistics [29]:

(3) $$A_{n}^{2}=n\cdot \int_{-\infty }^{\infty }\frac{{\left[Q\left(y\right)-F\left(y\right)\right]}^{2}}{F\left(y\right)\cdot \left[1-F\left(y\right)\right]}\cdot dF\left(y\right)$$

where *Q*(*y*) is the empirical distribution and *F*(*y*) is the theoretical distribution.

The value obtained is a measure of the discrepancy between the empirical distribution and the considered theoretical distribution [30].

For the empirical distribution, the relation is [31]

(4) $$Q\left[y_{\left(i\right)}\right]=\left\{\begin{array}{l} 0,\;\mathrm{for}\;Y<y_{\left(1\right)}\\ \frac{1}{n},\;\mathrm{for}\;y_{\left(i\right)}<Y<y_{\left(i+1\right)},\;i=\bar{1,n-1}\\ 1,\;\mathrm{for}\;Y>y_{\left(1\right)} \end{array}\right.,$$

Then, the Anderson–Darling test statistic result is defined by the following relation [29]:

(5) $$A_{n}^{2}=-\sum_{i=1}^{n}\frac{2\cdot i}{n}\cdot \left\{\ln F\left(y_{\left(i\right)}\right)+\ln\left[1-F\left(y_{\left(n+1-i\right)}\right)\right]\right\}-n,$$

where *A_n_^2^* is the Anderson–Darling statistic, *n* is the sample size, *F(y)* is the cumulative density function for the specified distribution, and *i* is the *i^th^* sample.

From a qualitative point of view, reliability represents the ability of a product to function without defects during a certain time interval, under given conditions.

The quantitative assessment of reliability or one of its characteristics can be achieved by applying reliability indicators. An effective measurement of reliability cannot be achieved, but only an estimation of one of the characteristics. The estimated values of the reliability indicators are determined by the statistical processing of the experimental or simulated data.

The statistical estimation of the lifetime data aims to determine the distribution law associated with the duration of operation of two types of dies, with the reliability indicators constituting numerical characteristics of these random values.

Parametric methods require prior knowledge of the distribution law of the operating time until failure. Instead, knowledge of the statistical model is suitable for a reliability study of the analyzed product. In the study of the statistical models of a reliability analysis, it is necessary to determine the appropriate method for identifying the statistical distribution.

After identifying the appropriate statistical model, a parametric distribution analysis of reliability indicators can be carried out by graphical or analytical methods. In this study, in order to estimate the main reliability indices, maximum likelihood estimation (MLE) with a 95% confidence interval (CI) was applied.

Maximum likelihood estimation is an accurate and easy method to estimate lifetime distribution parameters. For a Weibull distribution, the main indices are given by the following equations [31]:

(6) $$f\left(t\right)=\frac{\beta }{\eta }{\left(\frac{t-\gamma }{\eta }\right)}^{\beta -1}e^{-{\left(\frac{t-\gamma }{\eta }\right)}^{\beta }},$$

(7) $$F\left(t\right)=1-e^{-{\left(\frac{t-\gamma }{\eta }\right)}^{\beta }},$$

(8) $$R\left(t\right)=e^{-{\left(\frac{t-\gamma }{\eta }\right)}^{\beta }},$$

(9) $$z\left(t\right)=\frac{\beta }{\eta }{\left(\frac{t-\gamma }{\eta }\right)}^{\beta -1},$$

where *β* is the shape parameter, *η* is scale parameter, and *γ* is location parameter.

Let *f*(*t*) be the probability density function (PDF) and *F*(*t*) the cumulative density function (CDF) for the chosen life distribution model. The likelihood function is given by the following [25]:

(10) $$L\left(t_{1},t_{2},\ldots ,t_{n},,\beta ,\eta \right)=\frac{\beta^{n}}{\eta^{n}}\prod_{i=1}^{n}{\left(\frac{t_{i}}{\eta }\right)}^{\beta -1}e^{-\sum_{i=1}^{n}{\left(\frac{t_{i}}{\eta }\right)}^{\beta }}.$$

The general mathematical technique for solving MLEs involves setting partial derivatives of *ln*(*L*), leading to

(11) $$lnL\left(t_{1},t_{2},\ldots ,t_{n},,\beta ,\eta \right)=nln\beta -n\beta ln\eta +\left(\beta -1\right)\sum_{i=1}^{n}lnt_{i}-\frac{1}{\eta^{\beta }}\sum_{i=1}^{n}lnt_{i}^{\beta }.$$

Maximum likelihood equations for the Weibull distribution become [25]

(12) $$\left\{\begin{array}{c} \frac{1}{\hat{\beta }}+\frac{1}{n}\sum_{i=1}^{n}lnt_{i}-\frac{\sum_{i=1}^{n}t_{i}^{\hat{\beta }}lnt_{i}}{\sum_{i=1}^{n}t_{i}^{\hat{\beta }}}=0\\ {\hat{\eta }}^{\hat{\beta }}=\frac{1}{n}\sum_{i=1}^{n}{t_{i}}^{\hat{\beta }} \end{array}\right..$$

## 3. Results and Discussion

### 3.1. Reconditioned Dies by Welding Deposition 

**Figure 4:**
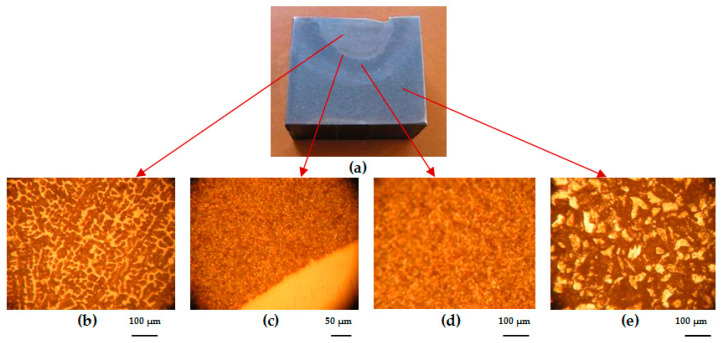
Macro- and microstructural analysis images of the loaded area: (**a**) plate macrostructure; (**b**) deposited material (DM), 100× magnification—casting structure for martensite with residual austenite and complex carbides evenly distributed; (**c**) transition area (TA), 50× magnification—complete melting without damage; (**d**) heat-affected area (HAZ), 100× magnification—martensite and bainite; (**e**) base material (BM), 100× magnification—recurrence structure.

On the experimental plates, cross-sections were taken of the deposits achieved. In specific areas of the deposits, the metallography was analyzed as follows: base metal (BM), transition zone (TZ), heat-affected zone (HAZ), and deposited metal (DM) (Figure 4). The preparation of metallographic samples for macro- and microscopic analysis was conducted in accordance with EN 1321:2000. The microstructural analyses were carried out on a Nikon (metallographic) optical microscope, the Eclipse MA 100 model.

**Figure 5:**
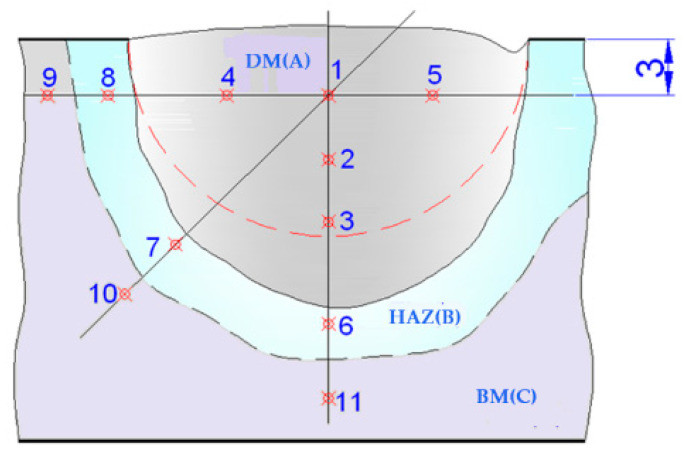
Specific areas for measuring the hardness of metal deposited by welding. Note: The dotted red line shows the semicircular milled groove for welding surface preparation. The points 1–11 indicate where the hardness was measured.

The hardness values in specific areas of the deposition welding (Figure 5), formed with the tested sample electrodes, are shown in Table 3. The hardness measurements (in deposition areas) were taken using an FM 700 micro durometer.

The data analysis results in Table 3 highlight that the hardness values of the deposited metal, in the welded state, were in accordance with product standards.

In order to establish the geometric configuration of the deposition support, the surface degradation (wear) manifested by cracks, fissures, and detachments of oxidized and damaged material, etc., was taken into account. Its profiling was carried out and showed that it formed a suitable basis for deposition.

**Figure 6:**
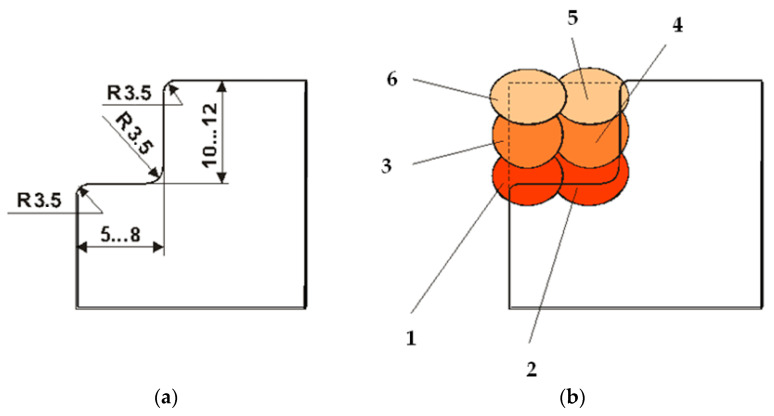
Profiling mode: (**a**) surface subjected to loading; (**b**) deposition order of weld beads (points 1–6).

It is recommended that the support should be prepared (profiled) according to Figure 6.

The technological process depends on the state of the dies (hardened or tempered) as well as the technical resources available.

An important role is played by the welding beads’ deposition order along the profiled area, which works to balance stresses, achieve a good penetration into the base metal between the layers, and avoid overheating. The mechanical processing required to obtain the nominal dimensions from technical documentation is minimal.

Then, the mechanical processing required to bring the loaded die to the operating level is carried out via specific procedures.

**Figure 7:**
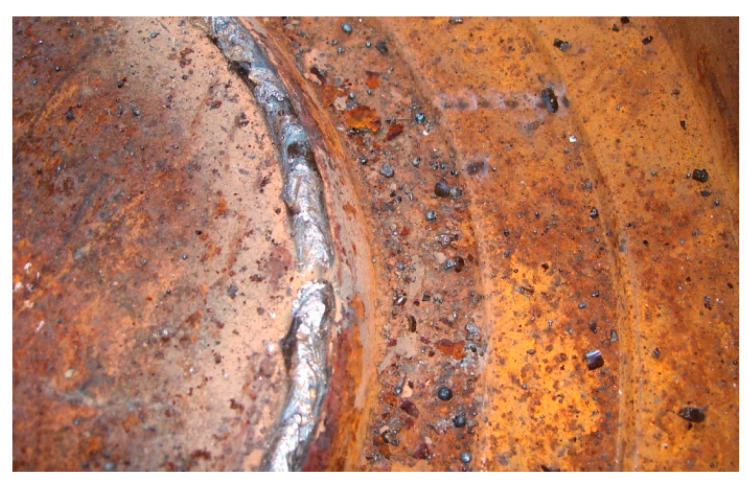
Half die reconditioned by loading with the experimental electrode.

In this study, after the analysis performed on the plates, six used dies were loaded by welding. Part of the loading of a half die is shown in Figure 7.

The half dies were tested in the manufacturing process of wagon wheels, and an increase in the operating time was observed. We found that, considering the maximal wear, half dies can undergo a maximum of five rounds of reconditioning and processing at the operating level, after which they should be completely removed from use.

### 3.2. Comparative Lifetime Analysis of Die Types

The inferential analysis in this study consisted of reliability indices’ estimation for different types of dies manufactured through various deposition welding processes.

The analyzed dies were as follows:

- Type I: Dies manufacturing by conventional process;
- Type II: Dies reconditioned by deposition welding with covered electrode.

Type I dies were manufactured by conventional processes that involve the following stages: the primary elaboration of the die blocks by forging, heat treatments, and mechanical processing to meet the dimensions in technical documentation.

Type II dies were failed and reconditioned dies, treated with deposition by electric arc welding using a specially designed covered electrode [16].

The lifetimes of Type I dies manufactured by conventional processes were recorded until the appearance of unacceptable defects (cracks, exfoliation, etc.). 

We analyzed the wear of the dies, which led to their removal from use after a relatively small number of manufactured wheels. The observed wear was mainly on an area of the active surface of between 2 and 6 mm. These dies were reconditioned by welding with the electric arc process using the specially covered electrode [16]. The reconditioned dies (Type II) were reintroduced into the wagon wheel manufacturing process, and we recorded the lifetimes until they were taken out of use.

**Figure 8:**
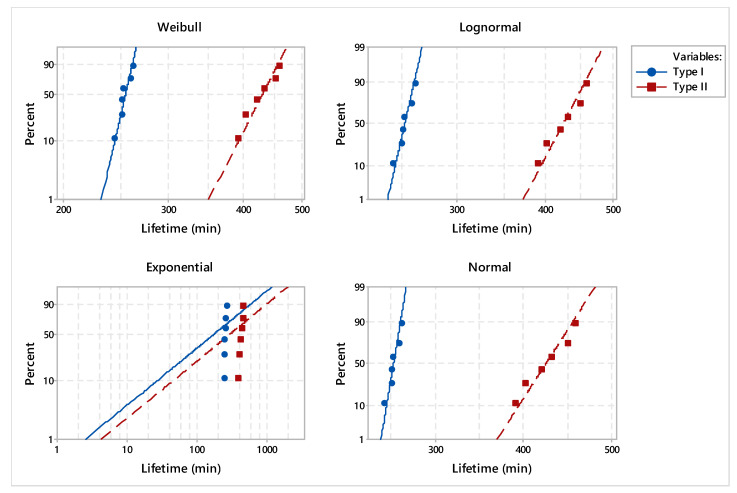
Probability plot for analyzed dies.

In order to validate the statistical distribution, the goodness of fit was applied. When analyzing the probability plots from Figure 8, it can be found that the points fall approximately on the straight line on the Weibull, with lognormal and normal repartitions. Furthermore, the Anderson–Darling goodness-of-fit values indicate which distribution best fits the data.

The estimated adjusted Anderson–Darling statistics (AD*) were comparable, with the only exception being the exponential distribution (Table 4). For the purpose of punctual estimation of the parameters’ distribution, we considered the Weibull distribution because it provided the best fit for the two types of dies.

To underline the differences between the two types of dies, the analysis consisted of comparing the main statistical parameters. Considering the Weibull distribution, the estimated shape and scale of the parameters are synthesized in Table 5. In addition, the distribution characteristics of dies manufactured by conventional processes and reconditioned by deposition welding process are presented in Table 6 and Table 7.

It can be underlined that shape and scale parameters for type II dies are significantly higher than in the case of type I dies. Regarding the mean time to failure (MTTF) for dies refurbished by deposition welding, it can be pointed out that the estimated value was 426.28 min compared to 252.82 min recorded for dies manufactured by conventional processes. In addition, the estimated values for the MTTF and median were similar in the case of the dies under study. Compared to type II, the standard deviation of type I dies was lower, indicating a statistical stability of lifetimes.

For the type II dies, the first quartile (Q1) was 411.76 min, the third quartile (Q3) was less than or equivalent to 444.1 min, and the estimated interquartile range was approximatively 33 min. Meanwhile, the type I milling teeth presented a first quartile (Q1) with a value of 249 min, a third quartile (Q3) that was smaller than or equivalent to 257.79 min, and an estimated interquartile range of approximately 8.75 min. 

**Figure 9:**
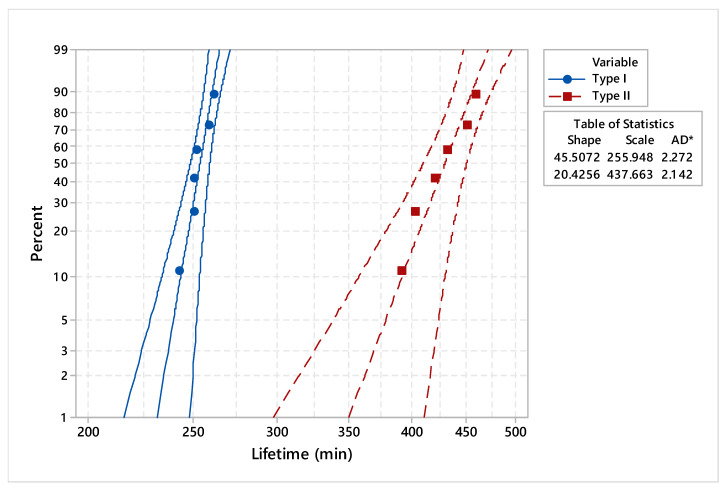
Comparative probability plot of analyzed dies.

**Figure 10:**
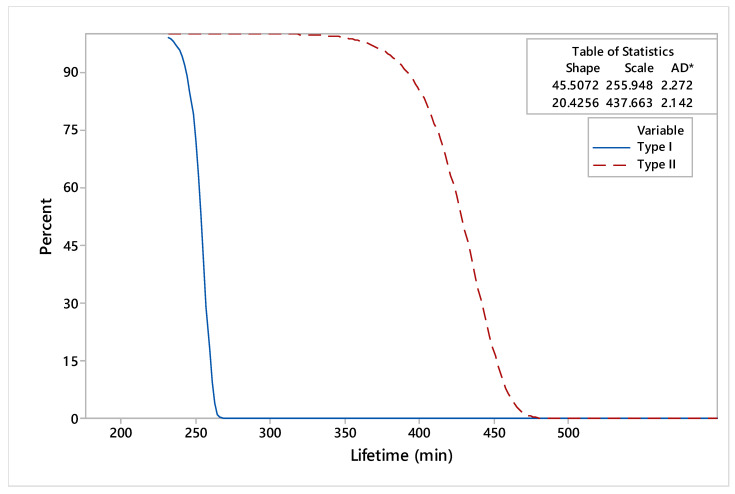
Comparative analysis of the reliability functions for the analyzed dies.

**Figure 11:**
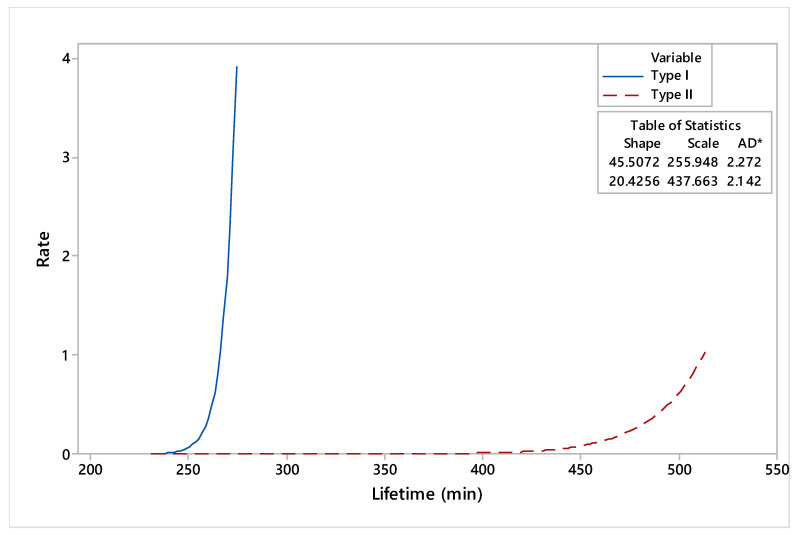
Comparative analysis of the hazard rate functions for the analyzed dies.

Probability plots for dies made by conventional and reconditioned processes are presented in Figure 9, with a 95% confidence interval (CI). The estimated reliability and hazard rate functions of the dies under study, based on lifetime data, are illustrated in Figure 10 and Figure 11.

The comparative analysis of reliability functions indicated that the maximum period of use for the type I dies was 269.3 min, while for the type II dies, it was 471.64 min. Moreover, based on the curves presented in Figure 11, there was an accentuated phenomenon of deterioration of the type I dies. Compared to type I, which showed an accentuated tendency of a hazard rate, the type II hazard rate tendency showed smooth variations over the working time.

The inferential statistical analysis of the two types of dies highlighted a considerable increase in the operating time for the dies reconditioned by deposition welding.

The deposition welding procedures were applied to parts that during functioning (exploitation) are subject to complex strains, in order to create self-protection systems against wear. These procedures present a series of advantages:

- They do not require complex, expensive, or complicated tools;
- The layers deposited by welding can have variable thicknesses;
- The preparation of the surface is not complicated, in most cases, being limited to simple washing and degreasing;
- They are productive, efficient, and have a low cost, and they can be mechanized and automated;
- Some parts that usually involve a large amount of labor and material can be manufactured from cheap materials, with the only exception being the active surfaces.

However, deposition welding processes also present certain disadvantages, such as the following:

- The deposited layer can be uneven, with the unevenness leading to an increase in the volume of subsequent mechanical processing;
- The high temperatures achieved in the seams during welding may change the structure of the base material of the part; therefore, after reconditioning, the parts will be subjected to thermal treatments, which implies additional energy and labor costs.

## 4. Conclusions

Dies are exposed to severe conditions—regular wear, impact, corrosion, thermal cycling, and high stress—causing deterioration [32]. As a result of this, according to Pecas et al. (2006), 80% of the dies used to manufacture automotive components undergo a repair/remanufacture process. Failures and wear are the main factors that affect service life and, in doing so, they can create unexpected high production costs [12]. In this context, the purpose of dies’ reconditioning, as presented in this paper, is to preserve specific characteristics for working conditions: high toughness, resistance to thermal fatigue, resistance to hot wear, and resistance to surface oxidation. 

The role of lifetime prediction is very important because it indicates the factors influencing the processes analyzed [33]. Lifetime analysis can be considered a method for improving dies’ design, manufacturing, and maintenance, which are the most effective techniques to prevent potential failure modes and effects. Die refurbishment consists of implementing a major overhaul of all die systems and restoring die surfaces in order to produce products within compliance specifications.

The most important conclusions resulting from our research and analysis are the following:

- The originality of this study is in the development of special additive materials for loading by welding, which, by deposition, confer superior wear resistance properties.
- The overall comparative analysis of the main reliability indicators highlighted that the dies reconditioned by deposition welding (type II) were superior to the dies manufactured by conventional processes (type I). The maximum recorded period of use for the type I dies was 269 min, while for the type II dies, it was 471 min. Also, he mean time to fail was 426 min for dies reconditioned by deposition welding compared to 253 min recorded for dies manufactured by conventional processes.
- In terms of the wear phenomenon, our study of the hazard rate functions of the dies under study underlined an accentuated phenomenon of damage for dies manufactured by conventional processes compared to smooth variations over the working time of dies reconditioned by deposition welding.
- The main objective of any organization is to obtain products in accordance with the specified requirements. In this sense, reconditioned dies could offer a cost-effective alternative to buying new.

## Figures and Tables

**Table 1:** Technical welding parameters.

Electrode Mark	Diameter (mm)	Welding Parameters			
		Welding Current (A)	Nat. Welding Current	Welding Temperature (°C)	Temperature between Coatings (°C)
EiCr2.5W4.5 V	4	130–160	CC+	400	400

**Table 2:** Chemical composition of the deposition metal.

Chemical Composition (%)								
C	Mn	Si	Cr	Mo	Ni	V	W	Co
0.2–0.5	-	1–1.5	2–3	max 1	max 1.5	0.4–1	3–5.5	-

**Table 3:** Hardness of specific loading areas.

Specific Areas	DM (A)					HAZ (B)			BM (C)		
Place No.	1	2	3	4	5	6	7	8	9	10	11
HRC	58.9	59.3	58.5	57.3	58.1	54.1	51.3	53.7	35.7	30.9	33.4

**Table 4:** Goodness of fit for analyzed dies.

Distribution	Anderson–Darling Test (AD*)	
	Type I	Type II
Weibull	2.27	2.14
Lognormal	2.25	2.14
Exponential	4	3.88
Normal	2.25	2.14

**Table 5:** Parameter estimates for analyzed dies.

Dies	Parameter	Estimate (min)	Standard Error (min)	95% Normal CI	
				Lower (min)	Upper (min)
Type I	Shape	45.50	14.38	24.48	84.57
	Scale	255.94	2.43	251.22	260.75
Type II	Shape	20.42	6.63	10.80	38.62
	Scale	437.66	9.24	419.91	456.17

**Table 6:** Characteristics of distribution for type I dies.

Parameter	Estimate (Min)	Standard Error (min)	95% Normal CI	
			Lower (min)	Upper (min)
Mean (MTTF)	252.82	2.86	247.27	258.49
Standard Deviation	7.01	2.13	3.86	12.74
Median	253.89	2.69	248.66	259.23
First Quartile (Q1)	249.03	3.68	241.90	256.37
Third Quartile (Q3)	257.79	2.32	253.27	262.38
Interquartile Range (IQR)	8.75	2.71	4.76	16.07

**Table 7:** Characteristics of distribution for type II dies.

Parameter	Estimate (min)	Standard Error (min)	95% Normal CI	
			Lower (min)	Upper (min)
Mean (MTTF)	426.28	10.60	406.10	447.58
Standard Deviation	25.88	7.78	14.36	46.65
Median	429.87	10.17	410.38	450.30
First Quartile (Q1)	411.76	13.72	385.71	439.57
Third Quartile (Q3)	444.71	8.91	427.57	462.54
Interquartile Range (IQR)	32.95	10.26	17.89	60.68

## Data Availability

Data are contained within the article.
